# Decision regret related to urinary diversion choices after cystectomy among Chinese bladder cancer patients

**DOI:** 10.1002/cam4.5281

**Published:** 2022-10-21

**Authors:** Yinmeng Hou, Yiqian Chen, Shicong Lai, Samuel Seery, Ling Wang, Xiaodan Li, Huixin Liu, Caipeng Qin, Wei Li, Xiangyun Lu, Chunxia Liu, Jia Wang, Tao Xu

**Affiliations:** ^1^ Urology Department Peking University People's Hospital Beijing China; ^2^ Lancaster University, Faculty of Health and Medicine Lancaster UK; ^3^ Office of Retirement Affairs Peking University People's Hospital Beijing China; ^4^ Nursing Department Peking University People's Hospital Beijing China; ^5^ Department of Academic Research Peking University People's Hospital Beijing China; ^6^ Urology Department The Affiliated Qingdao Central Hospital of Qingdao University, The Second Affiliated Hospital of Medical College of Qingdao University Qingdao Shandong China; ^7^ Department of Urology, Peking Union Medical College Hospital Beijing China; ^8^ Peking University Third Hospital Beijing China

**Keywords:** China, conflict, decision making, perception, quality of life, urinary bladder neoplasia

## Abstract

**Aim:**

To explore factors associated with decision regret after cystectomy among Chinese bladder cancer patients.

**Methods:**

This cross‐sectional study involved 112 patients, who had received radical bladder cancer resection. Participants were recruited from August 2021 until January 2022. The decision regret scale (DRS), decision conflict scale (DCS), and the Functional Assessment of Cancer Therapy–Bladder cancer (FACT‐BL) form were used to measure decision regret, decision conflict, and quality of life. Investigator‐designed items further explored perceptions involved in decision‐making participation and outcomes.

**Results:**

The average score for decision regret was 26.21 (SD 15.886), while decision conflict was 20.27 (SD 13.375) and quality of life was 94.74 (SD 20.873). 57.1% of our participants had a little knowledge about the quality of life of patients who chose an alternate urinary diversion method; however, only 13.4% reported having a clear understanding. In addition, 8.9%, 26.8%, and 36.6% thought that quality of life related to alternate decisions was poor, average, or good, respectively. Multiple regression analysis suggested that decision regret is associated with decision conflict, quality of life, and the perceptions that others (who took alternate urinary diversion decisions) had a better quality of life.

**Conclusion:**

Decision regret is common among Chinese bladder cancer patients, after cystectomy. The prevalence of regret appears to be much higher in Chinese bladder cancer patients compared to similar studies from other regions. Decisions in mainland China are often made by the treating physician or by family members which may cause more profound regret. However, education and economic status are positively related to higher levels of regret which creates questions around knowing, participation, and expectations, which must be explored.

## Graphical abstract

It is important to acknowledge that Chinese mainland culture and medical practice are distinct which presents a number of issues that need to be considered in more detail. Understanding how these distinctions influence regret and outcomes may enable us to develop patient education programs and to promote participation.

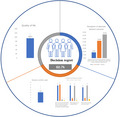

## 1 BACKGROUND

The gold standard treatment for myometrial invasive bladder cancer is radical cystectomy plus urinary diversion.^1^ At present, the most commonly implemented diversion methods are orthotopic bladder substitute (OBS), ileal bladder (IC), and ureterostomy, all of which have distinct advantages and disadvantages.^2^ The decision‐making process around which urinary diversion method to choose is rather complex. It is necessary to consider oncological characteristics, recurrence risk, complications, patients' condition, postoperative complications, postoperative Health‐related Quality of Life (HRQoL) and preferences.^3^ Each intervention is associated with risks, benefits, and harms. This makes patient‐practitioner consultations more complex and ultimately means that patients are more vulnerable to regret.

Decision regret is defined as the cognitive‐based negative emotion, experienced by individuals when they realize (or perceive) that an alternate choice would have resulted in an improved outcome.^4^ Decision‐making regret can be used in medicine to assess the quality of patients' decision‐making and is increasingly regarded as an important patient‐centered outcome.^5^ A systematic review in 2016 reviewed evidence around the degree of regret related to personal health decisions. The research included 59 studies of dozens of diseases such as breast cancer, prostate cancer, and lung cancer, and patients came from 11 countries such as the United States, Canada, Denmark, and the Netherlands. The results suggest that the average DRS score in each study was 16.5, and the proportion of patients with decision regret was between 4% and 20% based on a 25‐point scale in seven studies.^6^ This suggests that decision‐making regret is common in clinical settings and can have a detrimental impact. Research into decision regret is becoming more accepted and proving useful in prostate and breast cancer, although there have also been several related studies in the field of bladder cancer. For example, Check et al. published survey results that explored 192 bladder cancer patient regret at 6 and 12 months who were treated in the United States, postoperatively.^7^ However, the extent to which Check et als' findings can be extended to other countries, cultures, or medical settings is unclear. It is no secret that China has a unique cultural and political history, and therefore mainland Chinese people are also likely to process information and therefore regret in a distinct manner. By studying Chinese bladder cancer patients, we may be able to develop more effective, evidence‐based strategies for managing decision regret and for psychological well‐being, generally.

## 2 THE STUDY

### 2.1 Aim

To investigate factors associated with levels of regret among Chinese bladder cancer patients who underwent cystectomy.

### 2.2 Design

This is a multicenter, cross‐sectional exploratory study.

### 2.3 Participants

The population sample was obtained through urology departments in 31 tertiary care hospitals in mainland China. The people in charge of each department recruited patients through group messaging and by direct telephone contact. Inclusion criteria were: (i) patients above 18 years of age; and (ii) patients who received cystectomy with urinary diversion. Exclusion criteria included: (i) patients with underlying, diagnosed mental health issues, or severe cognitive impairment; (ii) complications such as related organ damage or other serious chronic disease/s; (iii) those with distant metastasis; and (iv) those with primary tumors in other organs.

### 2.4 Data collection

Data were collected from August 2021 until January 2022. All investigators were trained before data collection commenced. Investigators followed eligibility criteria strictly to identify possible participants. Once identified, investigators explained the purpose and requested written informed consent, before granting access. An electronic version of the questionnaire was distributed to all participants. After introducing the rules of completion to participants, they were asked to complete the form themselves. For illiterate participants, responses to questionnaires were recorded directly by investigators who read each item clearly and recorded answers.

### 2.5 Instruments

The key variables were collected, including sociodemographics, decision regret, decision conflict, Quality of Life (QoL), perceptions around alternate decision outcomes, and information around the decision‐making process.

A Chinese version of the decision regret scale (DRS) was used to assess the degree of decision regret. The DRS was validated with 704 inpatients and had internal consistency (*α* = 0.74) with test–retest reliability (intraclass correlation coefficient [ICC] = 0.71) which were considered “acceptable”.^8^ The scale includes five items and utilizes a five‐point Likert scale. Total scores ranged from 0 to 100. The higher the score, the greater the degree of decision‐making regret, with lower than 25 points representing relatively minor regret.

The Decision conflict scale (DCS),^9^ was compiled by O'Connor et al. and is the most widely used decision conflict assessment tool. Again, a Likert scale is used to generate scores of between 0 and 100. The higher the score, the more severe the decision conflict. A Chinese version of the DCS was used in this study. This version was verified in a study of 250 rectal cancer patients. The content validity index was 0.989. Cronbach's *α* coefficient was 0.886, and Cronbach's *α* of each dimension was 0.706–0.911.

The FACT‐BL^10^ consists of 39 items, including physical status (C7), social/family status (C7), emotional status (C6), and functional status (C7), plus 12 specific bladder cancer templates. Scores for each item were assigned using 0–4. The higher the score, the better the perceived QoL. The Chinese version of FACT‐BL was validated in 365 bladder cancer patients, and the results highlighted a Cronbach's α coefficient of 0.836, with each dimension between 0.772 and 0.918.

The cognitive element involved in perceiving “other decision outcomes” was measured using two investigator‐designed questions:

1. Do you understand the quality of life of patients who underwent an alternate urinary diversion surgery?
2. What do you think of the quality of life of patients who have undergone an alternate urinary diversion operation?

The decision‐making process was also explored using the following, investigator‐designed questions:

1. How do you learn information about the three interventions, such as operation methods, advantages and disadvantages, etc.?
2. Whose opinion was considered first through treatment consultations?
3. Who made the urinary diversion choice?

### 2.6 Ethical considerations

The ethics committee at Peking University People's Hospital reviewed and approved this study (reference number: 2021PHB235‐001). Prior to commencing the survey, researchers were provided study details. Participation was on a voluntary basis and participants could withdraw at any time, without impacting patients.

### 2.7 Data analysis

SPSS (version 26.0) software was used for data entry and analysis. Data were described and analyzed using means with corresponding standard deviations (SD), frequencies and composition ratios, and with Pearson's analysis, standard T‐tests, and variance analysis. Multiple linear regression analysis was used to explore the influencing factors of decision regret. The threshold for statistical significance was set at *p* < 0.05.

## 3 RESULTS

### 3.1 Characteristics of the participants and the relationships with decision regret

During the recruitment phase, a total of 141 patients agreed to participate although only 112 completed the questionnaire. The effective rate of the questionnaire was 100%. Most of the participants were men, relatively old, often retired, not well‐educated, low‐income, married, and had received a ureterostomy (Table 1). In terms of demographics, only level of education (F = 737.801*, p* = 0.03) and monthly family income (F = 6.525*, p* < 0.001) could be considered statistically significant factors influencing decision regret.

**TABLE 1:** Demographics and the relationships with decision regret

Variables		*n* (%)	Decision regret ($\bar{x}$±s)	F/t	*p*
Age (years)	<44	21 (18.8)	31.90 ± 10.895	2.245	0.084
	45–59	33 (29.5)	13.863 ± 2.413		
	60–74	44 (39.3)	15.828 ± 2.386		
	>75	14 (12.5)	23.323 ± 6.233		
Gender	Male	84 (75)	25.18 ± 16.307	−1.187	0.238
	Female	28 (25)	29.29 ± 14.383		
Education	Junior high school or below	34 (30.4)	20.29 ± 13.759	737.801	0.030
	High school	34 (30.4)	27.50 ± 16.663		
	Junior college	18 (16.1)	26.11 ± 14.303		
	Bachelor's degree or above	26 (23.2)	32.31 ± 16.627		
Marital status	Unmarried, divorced, or widowed	13 (11.6)	25.00 ± 14.434	0.084	0.773
	Married	99 (88.4)	26.36 ± 16.128		
Working status	On the job	39 (34.8)	28.33 ± 10.962	0.532	0.589
	Retire	60 (53.6)	25.08 ± 17.933		
	Unemployed	13 (11.6)	25.00 ± 18.708		
Average monthly household income (yuan)	<1000	15 (13.4)	22.67 ± 15.796	6.525	0.000
	1000–4999	42 (37.5)	19.40 ± 14.659		
	5000–9999	26 (23.2)	33.65 ± 15.333		
	≥10,000–100,000	29 (25.9)	31.21 ± 14.057		
Payment method	Public medical treatment	111 (99.1)	26.04 ± 15.856	1.418	0.236
	Completely at one's own expense	1 (0.9)	45.00 ± 0		
Operation mode	Orthotopic neobladder	22 (19.6)	20.91 ± 14.362	1.557	0.215
	Ileal bladder	38 (33.9)	27.11 ± 15.796		
	Ureterostomy	52 (46.4)	27.79 ± 16.372		

### 3.2 Decision regret, decision conflict, and quality of life

Table 2 presents mean scores for decision regret, decision conflict, all dimensions and total scores for quality of life. 60.7% had a score for decision regret ≥25, which represents moderate and severe decision regret.

**TABLE 2:** Decision regret, decision conflict, and quality of life

Items		($\bar{x}$±s)	*n* (%)	*n* (%)
Decision regret	Total scores	26.21 ± 15.886	Decision regret<25	Decision regret ≥25
			44 (39.3)	68 (60.7)
Decision conflict	Total scores	20.27 ± 13.375		
Quality of life	Total scores	94.74 ± 20.873		

### 3.3 Correlations between decision conflict, quality of life, and decision regret

Decision conflict positively correlates with decision regret, the total score for quality of life negatively correlates with decision regret and decision conflict. Also, the correlation coefficient between decision‐making conflict and quality of life was less than 0.7 (Table 3).

**TABLE 3:** Correlations between decision conflict, quality of life, and decision regret

Variables	Decision regret	Decision conflict
Decision conflict	0.707^a^, ^b^	—
Quality of life	−0.586^a^, ^b^	−0.501^a^, ^b^

^a^ Correlation is significant at 0.01 level.

^b^ Correlation is significant at 0.05 level.

### 3.4 Perception of alternate decision outcomes and interactions with regret

Most patients reported having some knowledge about the quality of life of patients (who chose an alternate urinary diversion method) and thought they had a better quality of life, which was significantly associated with decision regret (Table 4). It is worth noting that 31 patients were treated as missing at this analytical stage because they said they simply did not know about other patients' circumstances.

**TABLE 4:** Perception of alternate decision outcomes and its relation to decision regret

Item		*n* (%)	Decision regret ($\bar{x}$±s)	F/t	*p*
Do you understand the quality of life of patients who underwent alternate urinary diversion surgery?	Do not understand	33 (29.5)	29.85 ± 16.746	5.007	0.008
	Some understanding	64 (57.1)	26.95 ± 15.135		
	Very understanding	15 (13.4)	15.00 ± 12.677		
What do you think of the quality of life of patients who have undergone an alternate urinary diversion operation?	Bad	10 (8.9)	16.675 ± 5.273	0.251	0.778
	Average	30 (26.8)	16.795 ± 3.066		
	Good	41 (36.6)	15.005 ± 2.343		

### 3.5 Decision participation processes and its relationship with decision conflict

54.5% did not know relevant information about the three operations. For 45.5% of this sample, the doctors' opinion was considered first, and for 49% treatment choices were decided entirely by doctors or their families. Decision process factors appear to significantly relate to decision conflict (Table 5).

**TABLE 5:** Decision participation process and its relation with decision conflict

Items		*n* (%)	F/t	*p*
How do you learn information about the three interventions, such as operation methods, advantages and disadvantages, etc.?	No one told me	2 (1.8)	5.280	0.002
	Found information myself	5 (4.5)		
	Doctor	51 (45.5)		
	Family	54 (48.2)		
Whose opinion was considered first?	Doctor's	51 (45.5)	3.395	0.021
	My own	50 (44.6)		
	Family's	9 (8)		
	Others	2 (1.8)		
Who made the urinary diversion choice?	Doctor	45 (40.2)	3.304	0.031
	Myself	51 (45.5)		
	Family	10 (8.9)		
	Others	6 (5.4)		

### 3.6 Multivariate analysis

Variables with significant differences under univariate and correlation analysis were included in stepwise, multiple linear regression analysis as dependent variables. The results showed that decision conflict (*β* = 0.611*, p* < 0.001), quality of life (*β* = −0.286*, p* < 0.001) and the perception that other patients had a better quality of life when they chose an alternate urinary diversion method (*β* = 0.207*, p* < 0.01) were all significant predictors of decision regret. These factors explained 60.1% of the decision regret recorded (Table 6).

**TABLE 6:** Multivariate regression model for decision regret

Predictors	*R* ^2^	Adjusted *R* ^2^	B	SE	Beta	*t*	*p*	*VIF*
Decision conflict	0.500	0.496	0.726	0.085	0.611	8.552	<0.001	1.422
Quality of life	0.572	0.564	−0.218	0.053	−0.286	−4.111	<0.001	1.394
Good^a^	0.612	0.601	6.797	2.031	0.207	3.346	0.001	1.066

*Note*: β, standardized coefficients. Dependent variable: decision regret.

^a^ Perception that other patients had a better quality of life who chose an alternate urinary diversion method.

## 4 DISCUSSION

The aim of this study was to add reliable evidence to the decision‐regret knowledge base. By studying Chinese bladder cancer patients, it was also hoped that we could gain insight to promote best practice around the world. This study included 112 patients with bladder cancer from five provinces in mainland China. The results suggest that regret is widespread among the patients in China. In this study, 60.7% of the patients had some form of regret, and the average decision regret of 26.21 (SD = 15.886), much higher than that in Check et als' study where the average decision regret at six and 12 months after operation were 15.5 (SD = 18.3) and 13.8 (SD = 19.9).^7^

In this study, education and monthly family income were influencing factors in decision regret. We found that patients with an undergraduate degree or higher, and those who had a monthly family income greater than 5000 yuan (equivalent to approximately 785 USD, January 2022), had a higher level of decision‐regret. One possible explanation can be found in Noguera et als' study,^11^ which investigated 387 cancer patients and found that more highly educated patients were more likely to actively participate in the decision‐making process. In this study, due to the “special” decision‐making environment in China, many patients did not fully participate in the decision‐making process. 55.4% of the patients said that the opinions of other people were given priority, and 54.5% did not make the surgical decision by themselves.

In studies which explored different clinical backgrounds, factors related to decision‐making process, such as decision conflict and decision participation, appear significantly related to decision‐making regret.^12^, ^13^, ^14^ We established decision conflict as an independent factor for decision regret, which can be used to explain 50% of all regret. However again, Chinese society and culture are nuanced and in some ways quite different from other more Westernized societies. For example, in China, they have what is known as a “family consent notification” which is used to withhold cancer information from patients. This is commonly used for elderly, untreatable patients who would not benefit from knowing the severity of their condition. In more Westernized societies, this may be considered unethical or at least a breach of privacy; however, some Chinese believe that knowing may be related to the rapidity of decline.

Liu et al. studied 124 cancer patients in China and found that only 37.9% knew they actually had cancer.^15^ At the same time, and this is age‐related, most family members do not want their elderly relatives to participate in the decision‐making process, and therefore treatment‐related information is not fully divulged through clinical consultations, as the result of this study showed that 54.5% of the patients did not get information about the operation directly from the doctors, but from their families. From a Western clinical perspective, this again may appear unethical but it is also fair to say that both traditional/modern medicines or Western/Eastern approaches incorporate beliefs. Placebo and nocebo effects have been extensively reported and studied so there are benefits to understanding both perspectives on healthcare. In a survey of 180 Chinese cancer patients, researchers report that it is common practice for doctors to discuss treatment plans with families.^16^ In the same study, 55% of patients were not directly informed of the advantages and disadvantages of each decision, and 50% also said that the opinions of doctors and family members were considered first which is consistent with the findings of our study. Patient preferences are not always consistent with clinicians or, in this instance, with family members and therefore may increase the risk of encountering regret.^17^

The total quality of life of patients in this study was 94.74 with an SD of 20.873. This is lower than 124 (SD = 15) reported in the Allareddy et als' study and the quality of life observed in Tsaturyan et al. study (mean 106.3, SD = 20.4).^18^, ^19^ Quality of life is undoubtedly an independent influencing factor involved in decision regret. Although, a causal relationship between decision regret and quality of life has not been established. Some suggest that regret may come from the fact that patients are aware of the result of an alternate choice.^20^ For most medical decisions, the results of alternative choices are unknown although, this study appears to confirm that patients who perceive other patients who chose a different urinary diversion method have a better quality of life, have more regret than those who believe that patients who make other choices have a similar or poorer quality of life. To some extent, regret and how one copes with regret may perpetuate depression and anxiety which are common comorbidities in those with bladder cancer who received cystectomy. Even though it is beyond the remit of this article to discuss these in any great detail, it is necessary to conduct more research to disentangle elements which directly relate to survivorship. The difficulty here is to explore established concepts without minimizing their meaning or importance.

Before moving onto recommendations, it is important we briefly mention some of the major limitations of this study. First, we would have liked to explore levels of optimism, pessimism, depression, and anxiety in relation to the decision‐making process and regret. Also, potentially influential disease‐specific factors were not available. Also, participants in our research are convenient samples and come from tertiary care hospitals, so the representativeness is limited.

## 5 CONCLUSION

Decision regret is common among Chinese bladder cancer patients, after cystectomy. The prevalence of regret appears to be much higher in Chinese bladder cancer patients compared to similar studies from other regions. Decisions in mainland China are often made by the treating physician or by family members which may cause more profound regret. However, education and economic status are positively related to higher levels of regret which creates questions around knowing, participation, and expectations, which must be explored.

## AUTHOR CONTRIBUTIONS

**Yinmeng Hou:** Data curation (equal); investigation (equal); methodology (supporting); project administration (equal); writing – original draft (lead); writing – review and editing (supporting). **Yiqian Chen:** Data curation (equal); investigation (equal); methodology (equal); project administration (equal). **Shicong Lai:** Methodology (supporting); writing – original draft (supporting); writing – review and editing (lead). **Samuel Seery:** Writing – original draft (supporting); writing – review and editing (equal). **Ling Wang:** Investigation (supporting); resources (supporting). **Xiaodan Li:** Investigation (supporting). **Huixin Liu:** Methodology (equal). **Caipeng Qin:** Writing – original draft (supporting). **Wei Li:** Investigation (supporting). **Xiangyun Lu:** Investigation (supporting). **Chunxia Liu:** Investigation (supporting). **Jia Wang:** Conceptualization (supporting); resources (equal); supervision (supporting). **tao xu:** Conceptualization (lead); methodology (equal); resources (equal); supervision (lead).

## RESEARCH INVOLVING HUMAN PARTICIPANTS AND/OR ANIMALS

This is a cross‐sectional survey involving human participants. The Ethics Committee at Peking University People's Hospital reviewed and approved this study (reference no. 2021PHB235‐001).

## CONSENT TO PARTICIPATE

This study involved distributing questionnaires via the Internet rather than face‐to‐face data collection. The first page of the questionnaire outlines the research background, purpose, and explains the methods involved in this study. Voluntarily participation and right to withdraw at any time were emphasized. Written informed consent was required before participating in this study.

## Data Availability

Data from this this will be made available by the corresponding author upon reasonable request.
