# Erratum

**DOI:** 10.1002/cam4.5814

**Published:** 2023-10-18

In the article by Hou Y et al.,^1^ Jia Wang was designated as a co‐corresponding author and Funding Information was added as follows:

## FUNDING INFORMATION

National Key Research and Development Program of China (no. 2018YFA0902802).
